# Lung Function before and Two Days after Open-Heart Surgery

**DOI:** 10.1155/2012/291628

**Published:** 2012-08-08

**Authors:** Charlotte Urell, Elisabeth Westerdahl, Hans Hedenström, Christer Janson, Margareta Emtner

**Affiliations:** ^1^Physiotherapy, Department of Neuroscience, Uppsala University, 751 24 Uppsala, Sweden; ^2^School of Health and Medical Sciences, Örebro University, 701 82 Örebro, Sweden; ^3^Clinical Physiology, Department of Medical Sciences, Uppsala University, 751 05 Uppsala, Sweden; ^4^Respiratory Medicine and Allergology, Department of Medical Sciences, Uppsala University, 751 85 Uppsala, Sweden

## Abstract

Reduced lung volumes and atelectasis are common after open-heart surgery, and pronounced restrictive lung volume impairment has been found. The aim of this study was to investigate factors influencing lung volumes on the second postoperative day. Open-heart surgery patients (*n* = 107, 68 yrs, 80% male) performed spirometry both before surgery and on the second postoperative day. The factors influencing postoperative lung volumes and decrease in lung volumes were investigated with univariate and multivariate analyses. Associations between pain (measured by numeric rating scale) and decrease in postoperative lung volumes were calculated with Spearman rank correlation test. Lung volumes decreased by 50% and were less than 40% of the predictive values postoperatively. Patients with BMI >25 had lower postoperative inspiratory capacity (IC) (33 ± 14% pred.) than normal-weight patients (39 ± 15% pred.), (*P* = 0.04). More pain during mobilisation was associated with higher decreases in postoperative lung volumes (VC: *r* = 0.33, *P* = 0.001; FEV_1_: *r* = 0.35, *P* ≤ 0.0001; IC: *r* = 0.25, *P* = 0.01). Patients with high BMI are a risk group for decreased postoperative lung volumes and should therefore receive extra attention during postoperative care. As pain is related to a larger decrease in postoperative lung volumes, optimal pain relief for the patients should be identified.

## 1. Introduction

Reductions in lung volumes and oxygenation are common during the initial period after open-heart surgery. The effects of the median sternotomy, hypothermia for myocardial protection, dissection of the internal mammary artery, and the use of cardiopulmonary bypass negatively influence lung function [1–4].

In comparison to preoperative values, a mean reduction of forced vital capacity (FVC) and forced expiratory volume in 1 second (FEV_1_) are reported to be 40–50% on the first-to-third postoperative days [5]. On the second postoperative day, a mean reduction of 63% in vital capacity (VC), compared to preoperative values, is reported, and lung volumes can remain depleted for three-to-four months after surgery [6–8]. Postoperative atelectasis are common in all patients undergoing open-heart surgery and the reduced lung volumes contribute to impaired gas exchange [9]. An inverse correlation between atelectatic area and arterial oxygenation (PaO_2_) is reported on the first [10] and second [11] postoperative days after open-heart surgery. In addition, chronic obstructive pulmonary disease (COPD), general health status, smoking history, and age are factors associated with increased risk for impaired lung volume after surgery [12, 13].

Interventions such as postoperative breathing exercises, effective coughing techniques, early mobilisation, and inspiratory muscle training are used by physiotherapists to limit lung volume decreases and atelectasis and to increase oxygenation after open-heart surgery [12, 14–17]. Oxygenation improves in patients performing a high rate of postoperative breathing exercises [18]. As pronounced lung volume impairment occurs in the immediate period, measured by spirometry [18], the factors that might affect lung volumes on the second postoperative day after open-heart surgery would be of the greatest value to investigate.

The aim of this study was to investigate the pre-, peri-, and postoperative factors influencing lung volumes, measured by spirometry, on the second postoperative day after open-heart surgery. The hypothesis was that age, obesity, smoking, airflow obstruction, and pain influence lung volumes negatively in the immediately period after surgery.

## 2. Methods and Materials

### 2.1. Sample

Adult patients (>18 years) undergoing non-emergency open-heart surgery (coronary artery bypass grafting (CABG) or valve surgery) during 2007-2008 at a University hospital in Sweden were potentially eligible for this study. Patients with angina at rest preoperatively, postoperative artificial ventilation for more than 15 h or continuous positive airway pressure (CPAP) treatment, or patients who had difficulty understanding the Swedish language were not included. Patients, who could not conduct spirometry correctly, were excluded. The regional ethical review board in Uppsala, Sweden, approved the study. All patients included in the study provided informed consent.

### 2.2. Surgery and Postoperative Care

The patients underwent aortic, mitral, or tricuspidal valve replacement or CABG. The surgical approach was through median sternotomy, and cold-blood cardioplegia was used. All patients received general anaesthesia and the inspired oxygen fraction (FIO_2_) was between 35–60%. The pericardium, the mediastinum, and occasionally one or both pleura were drained approximately 36 hours after surgery. The patients were extubated when they had resumed normothermia, had no severe bleeding on drainage, and were haemodynamically stable and able to breathe adequately. After extubation, all patients received supplemental oxygen for maintaining arterial oxygen saturation (SaO_2_) above 90%.

Pain relief was administrated to all patients according to standard routines at the clinic. On the day of surgery, 1 g paracetamol was administrated intravenously four times a day and a morphine infusion of 1 mg/ml, 0.5–2 ml/h. On the first and second days after surgery, all patients were given 1 g paracetamol orally four times a day and 5–10 mg oral morphine three to four times a day.

The patients performed postoperative breathing exercises and were mobilised as early as possible. Patients were mobilised to sitting on the bedside as soon as possible after extubation. Standing and mobilisation to an armchair was recommended on the first postoperative day. The physiotherapist instructed the patients daily on coughing techniques and exercises of the shoulder girdle. All patients performed hourly deep breathing exercises with a positive expiratory pressure (PEP) of 10–15 cm H_2_O, during the first two postoperative days, as described in detail in a previously study [18]. 

### 2.3. Measurements

Demographic data were extracted from the medical records. Lung volumes were measured by spirometry preoperatively and after removing the drains the morning of the second postoperative day (Cardio Perfect dynamic spirometry, Welch Allyn, NY, USA). The patients were in a sitting position and used a nose clip. The variables measured were VC, FVC, FEV_1_, and inspiratory capacity (IC). The highest value of three technically satisfactory manoeuvres was retained for VC, FVC, and FEV_1_, and a mean value for IC was calculated. The highest value of VC and FVC was used for VC. Predicted values for lung function were related to sex, age, and height according to Hedenstrom et al. [19, 20]. Patients with preoperative FEV_1_/(F)VC < 0.70 were defined as having airflow obstruction.

Pre- and postoperative lung volumes were expressed as a percent of predictive values and the decrease in lung volumes on the second postoperative day were expressed as reduction in percent (preoperative value − postoperative value/preoperative value ×100). Pain from the median sternotomy incision was quantified with a numeric rating scale (NRS) (0 = no pain to 10 = the worst imaginable pain) [21] at rest, during PEP use, while taking a deep breath, when moving from lying to sitting, and while coughing on the second postoperative day.

### 2.4. Statistical Analyses

Univariate and multivariate analyses were used to investigate the factors that might influence postoperative lung volumes and decrease in lung volumes. The dependent variables were VC, FEV_1_, and IC. The independent preoperative variables were gender, age, body mass index (BMI), airflow obstruction, New York Heart Association (NYHA) classification, diabetes, current smoking, and pack years. The independent perioperative variables were type of surgery, anaesthesia time, operation time, and pleura entering. The postoperative independent variables were postoperative weight gain (hyperhydration), time in the intensive care unit (ICU), total time in thoracic surgery ward, and different rates of deep breathing exercises (10 and 30 every hour awake) [18]. The associations in the univariate analyses were calculated with Spearman rank correlation test or Pearson correlation test. The independent variables associated with impairment in VC, FEV_1_, or IC in the univariate analyses with a *P* ≤ 0.1 were included in the forward multiple regression analyses.

Kruskal-Wallis test was used to test differences between different age groups and pain from the sternotomy. Analysis of variance (ANOVA) was used to test differences between different age groups regarding postoperative percent of predicted lung volumes and to analyse differences in decrease of lung volumes, between pain groups (measured with numeric rating scale NRS). Unpaired students *t*-test was used to test gender differences and differences between levels of BMI (≤25, >25) in postoperative percent of predicted values of lung volumes. The association between pain and decrease in postoperative lung volumes was analysed by Spearman rank correlation test. The level of significance was set at *P* < 0.05.

## 3. Results

From the 216 patients eligible for this study, four declined participation. Of these, 105 patients were not included, 31 patients had postoperative artificial ventilation >15 h or CPAP treatment, and 74 patients did not conduct the postoperative spirometry correctly due to fatigue. This resulted in a study population of 107 individuals with a mean age of 68 ± 9 years (37 to 86 years), and of which 80% were male. 50% of the patients had CABG surgery, 19% were classified as NYHA IIIB-IV, 61% with a BMI higher than 25 kg/m^2^, and 8% were smokers (Table 1).

### 3.1. Pre- and Postoperative Lung Volumes

**Figure 1:**
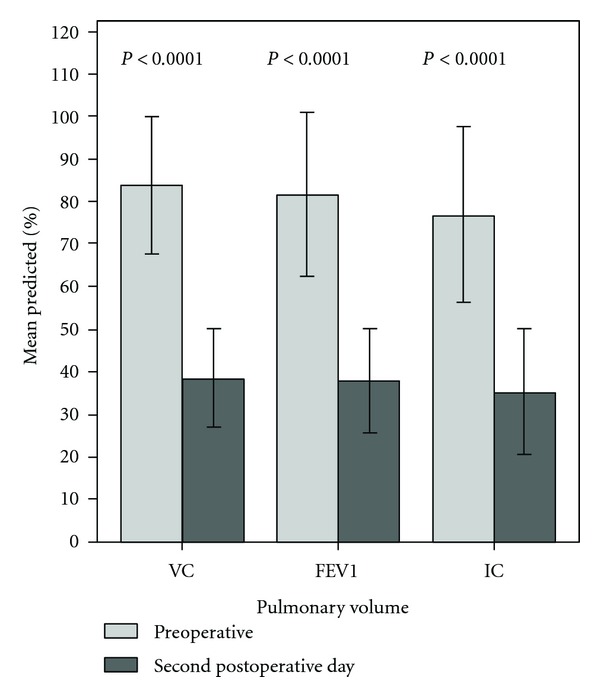
Lung volumes, percent of predicted values, preoperatively and on the second postoperative day, mean and SD, (*n* = 107). VC: vital capacity, FEV_1_: forced expiratory volume in 1 second, and IC: inspiratory capacity.

Preoperative lung volumes were in accordance with or just below predictive values, whereas postoperative volumes were less than 40% of predictive values (Figure 1). The mean decreases in lung volumes were 53 ± 14% for VC, 52 ± 18% for FEV_1_ and 53 ± 16% for IC (Figure 1). Postoperative mean volumes (in litre) were 1.64 ± 0.50 for VC, 1.20 ± 0.41 for FEV_1_, and 1.40 ± 0.47 for IC. The values for postoperative VC in different age groups were 1.36 ± 0.36 litre (37–60 years), 1.66 ± 0.54 litre (61–70 years), 1.67 ± 0.48 litre (71–80 years), and 1.46 ± 0.54 litre (81–86 years). Postoperative IC was 1.46 ± 0.48 liter in males and 1.16 ± 0.36 liter in females (*P* = 0.02).

### 3.2. Postoperative Lung Volumes—Univariate Analyses

In the univariate analyses, low age was statically significantly associated with low postoperative VC (expressed as % pred.) (Table 2). Low age, high BMI, valve surgery, and more time in the ICU were associated with low postoperative FEV_1_, and male gender was significantly associated with low postoperative IC (Table 2).

### 3.3. Postoperative Lung Volumes—Stratified and Multivariate Analyses

**Figure 2:**
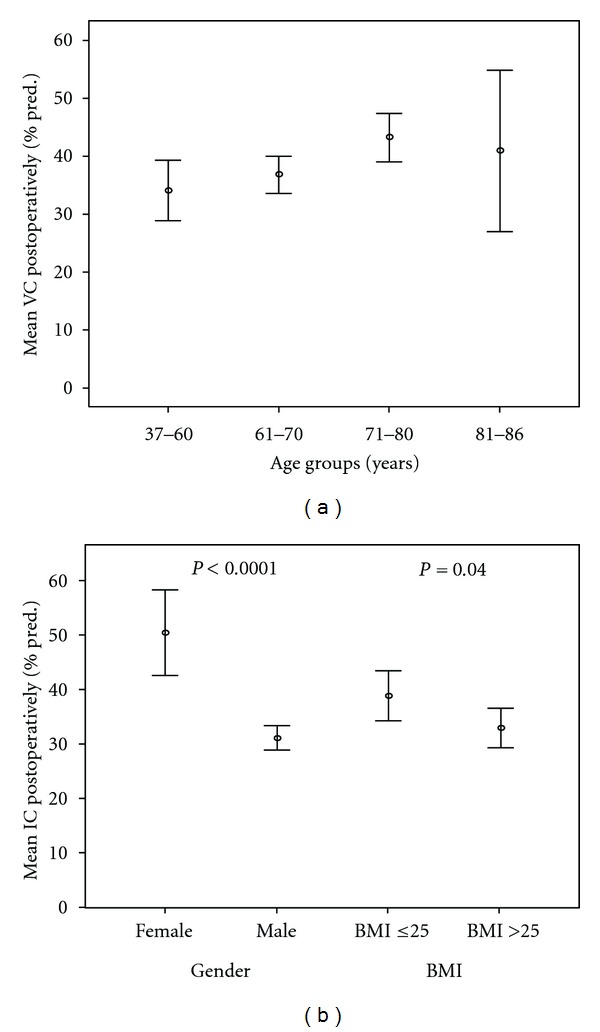
(a) Mean VC, 95% CI, postoperatively in percent of predictive values for patients in different age groups (*n* = 107; 37–60 years, *n* = 20; 61–70 years, *n* = 46; 71–80 years, *n* = 35; 81–86 years, *n* = 6);  *P* = 0.04 between all groups. (b) Mean IC, 95% CI, postoperatively in percent of predictive values for different genders and BMI. (*n* = 107, female *n* = 21, male *n* = 86, BMI ≤ 25 *n* = 42, BMI > 25 *n* = 65).

Low age was significantly associated with low postoperative VC (expressed as % pred.) in the multivariate analyses (Table 3). In the stratified analysis younger patients had lower postoperative VC than older ones: 34 ± 11% pred. (37–60 years), 37 ± 11% pred. (61–70 years), 43 ± 12% pred. (71–80 years), and 41 ± 13% pred. (81–86 years.) (Figure 2(a)). Male gender and high BMI were significantly associated with low postoperative IC (expressed as % pred.) in the multivariate analyses (Table 3). In the stratified analyses males had lower postoperative IC (31 ± 11% pred.) than females (50 ± 18% pred.), and patients with BMI > 25 had lower postoperative IC (33 ± 14% pred.) than patients with BMI ≤ 25 (39 ± 15% pred.) (Figure 2(b)).

### 3.4. Decrease in Lung Volumes—Univariate Analyses

In the univariate analyses diabetes was associated with significantly less decrease in lung volume, while having to spend more time in the ICU was associated with greater decrease in FEV_1_ (Table 4). Current smoking was associated with a smaller decrease in IC (Table 4).

### 3.5. Decrease in Lung Volumes—Multivariate Analyses

Smoking was the only independent variable associated with decrease in lung volume. Current smokers had less pronounced decrease in IC (adjusted difference −15.5 (−26.8, −4.20) % (B (95% CI)).

### 3.6. Postoperative Pain

**Figure 3:**
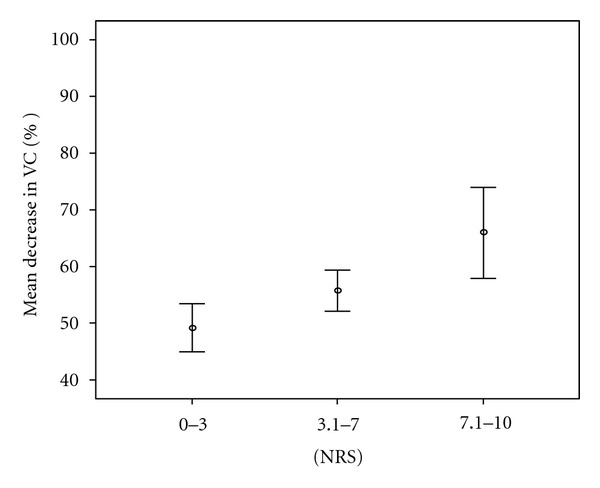
Mean VC, 95% CI, decrease in percent in relation to self-rated pain when moving from lying to sitting (*n* = 107, NRS 0–3.0 *n* = 52, NRS 3.1–7.0 *n* = 49, NRS 7.1–10.0 *n* = 6). NRS: numeric rating scale, 0 = no pain, 10 = the worst imaginable pain, VC: vital capacity,  *P* = 0.003 between all groups.

Postoperative pain from the sternotomy, as measured by NRS, was 1.0 ± 1.7 (median 0.2) at rest, 2.3 ± 2.1 (median 2.0) during PEP use, 3.1 ± 2.0 (median 3) while taking a deep breath, 3.5 ± 2.4 (median 3.8) when moving from lying to sitting, and 4.6 ± 2.2 (median 4.5) while coughing. Pain, from sternotomy when moving from lying to sitting, was in different age groups 4.7 ± 2.3 (median 5) (37–60 years), 3.2 ± 2.4 (median 3) (61–70 years), 3.2 ± 2.5 (median 3) (71–80 years), and 3.5 ± 2.1 (median 4.5) (81–86 years). The patients who rated their pain as 0–3 on NRS, when moving from lying to sitting, had less decrease in postoperative VC (49 ± 15%) compared to patients rating their pain as 3.1–7.0 (56 ± 12%) or 7.1–10.0 (66 ± 8%) (Figure 3). More pain during mobilisation (moving from lying to sitting) was associated with higher decreases in postoperative lung volumes (VC: *r* = 0.33, *P* = 0.001; FEV_1_: *r* = 0.35, *P* ≤ 0.0001; IC: *r* = 0.25, *P* = 0.01).

## 4. Discussion

Lung function was decreased by approximately 50% and the postoperative lung volumes were less than 40% of predictive values on the second postoperative day after open-heart surgery. Expressed as percent of predicted, younger patients had lower postoperative VC than older patients, males had lower postoperative IC than females, and patients with a high BMI had lower postoperative IC than normal weight patients. There was an association between more pain and more pronounced decreases in lung volumes postoperatively.

The decreased lung volumes were in accordance with previous studies. Nicholson et al. [5] reported a mean reduction of approximately 40–50% in FEV_1_ and FVC on the first and third postoperative day, and Matte et al. [6] reported a reduction of 53% (VC) on the first postoperative day and 63% on the second postoperative day. The causes of reduced lung volumes are probably multifactorial and may involve a combination of surgery, anaesthesia, immobilisation, and pain [22]. Reduced lung volumes affect gas exchange and an inverse correlation between atelectatic area and arterial oxygenation (PaO_2_) during the first postoperative days after open-heart surgery have been described [8, 10].

When lung volumes were expressed as percent of predicted value, younger patients had lower postoperative VC than older patients. However, when lung volumes were expressed in litre there were no differences between younger and older patients. One reason that younger patients seem to be more affected by surgery could be that they normally may use a greater range of motion in thorax during breathing compared to older ones. Another reason might be pain, as younger patients rate more pain from the sternotomy than older ones. Though males had lower IC (expressed as % pred.) compared to females this was probably not harmful because the values in litre were higher for males. Unfortunately there were only 20% females in this study, so we cannot draw any clear conclusions regarding gender differences.

Patients with a high BMI had significantly lower postoperative IC (expressed as % pred.) than the normal weight patients had. Obesity, even if it is mild, decreases lung volumes postoperatively [23, 24], and one reason for the lower IC is probably the flattened shape of the diaphragm and the mechanical pressure created by abdominal adiposity, which reduce the total space for the lungs. Atelectasis is more common in obese patients than in normal weight patients, and the time spent in the ICU and total hospital stay are longer for obese patients [23]. Thus, patients with high BMI are at increased risk of impaired pulmonary volumes after surgery.

Smoking and airflow obstruction were hypothesized as influencing the lung volumes negatively. In the multivariate analyses smokers had less pronounced decrease in IC and there was no difference in decrease in lung volume between patients with or without airflow obstruction. Smokers and patients with airflow obstruction are considered as risk patients for postoperative morbidity and mortality after CABG, even if the evidence is contradictory. Although a positive association between a diagnosis of COPD, or a low FEV_1_, and post-CABG mortality and morbidity are reported [25], the presence and worsening of airflow obstruction in patients with mild or moderate COPD is not associated with greater risk of mortality after CABG compared to patients with normal lung volumes [26].

There were lower levels of pain from the median sternotomy on the second postoperative day than presented in previous studies [1, 27, 28]. During rest the patients had a median value of less than 1 on NRS, that is, almost no pain and during mobilisation a median value of 3.8 on NRS. Postoperative pain may be associated with changes in thorax mechanics from the surgery, which in turn can influence the performance of deep breathing and effective cough [29]. Possible reasons for the lower levels of pain could be an individual pain relief strategy, deep breathing exercises with PEP directly after extubation, or early mobilisation. The association between decreases in lung volumes and high pain levels, that is, patients with more pain had the greatest decrease in lung volume, is in accordance with other results [22, 30].

There were some limitations to this study. Seventy-four patients were unable to perform postoperative spirometry due to fatigue and were not included in the analyses. The fatigue might have been due to the anaesthesia and the surgical procedure or because of pain relief postoperatively. These patients probably had further decreased lung volumes, but as they were unable to perform spirometry correctly, it was not possible to receive data. These patients could be a target group for future studies, and it is important to find a test to measure their lung function. As 8% of the patients were smokers in this study, the results on the basis on these patients should be taken with caution. The atelectatic area was not measured in this study: therefore the relation between the lung volumes and atelectatic area, which is clinically relevant, could not be studied. 

In conclusion there were large decreases in lung volumes two days after open-heart surgery. Patients with high BMI had lower postoperative lung volumes, which is an indication that these patients should receive extra attention during postoperative care. Postoperative pain was related to a larger decrease in postoperative lung volumes: therefore, it is important to determine optimal pain relief for the patient. 

## Figures and Tables

**Table 1:** Descriptive data of the study group (*n* = 107).

	*n* = 107
Male/female, %	80/20
Age, year	68 ± 9
NYHA I-IIIA/IIIB-IV/missing, %	67/19/14
BMI kg/m^2^	27 ± 4
BMI > 25 kg/m^2^, %	61
Smoker/former smoker/never smoked, %	8/41/51
Airflow obstruction, %	13
Diabetes, %	18
CABG/valve surgery %	50/50
Anaesthesia time, h	5.6 ± 1.4
Operation time, h	3.5 ± 1.1
Time in the ICU, h	53 ± 30

Data are presented in mean ± SD or %. BMI: body mass index, CABG: coronary artery bypass grafting, ICU: intensive care unit, NYHA: New York Heart Association. Airflow obstruction: defined as FEV_1_/(F)VC < 70%, valve surgery: aorta, mitralis, or tricuspidalis valve surgery.

**Table 2:** The variables associated with postoperative lung volumes (expressed as percent of predicted values), (*n* = 107). Univariate analyses.

Independent variables	Dependent variables		
	VC	FEV_1_	IC
	*B* (95% CI)	*B* (95% CI)	*B* (95% CI)
Males versus female	−4.90 (−10.4, 0.59)^†^	—	−19.4 (−25.4, −13.5)^∗^
Age/10 years	3.00 (0.53, 5.50)^∗^	3.03 (0.40, 5.70)^∗^	—
BMI weight (kg)/length m^2^	—	−0.78 (−1.42, −0.13)^∗^	−0.70 (−1.49, 0.08)^†^
CABG versus valve surgery	—	5.29 (0.58, 9.99)^∗^	—
ICU time/4 h	—	−0.34 (−0.66, −0.24)^∗^	—

VC: vital capacity, FEV_1_: forced expiratory volume in 1 second, IC: inspiratory capacity, BMI: body mass index, type of surgery (CABG: coronary artery bypass grafting, valve: aorta, mitralis, or tricuspidalis valve surgery), ICU: intensive care unit. ^∗^ *P* < 0.05, ^†^ *P* = 0.05–0.10, *B*: regression coefficient, CI: confidence interval.

**Table 3:** The variables associated with postoperative lung volumes (expressed as percent of predicted values), (*n* = 107). Multivariate analyses.

Independent variables	Dependent variables		
	VC	FEV_1_	IC
	*B* (95% CI)	*B* (95% CI)	*B* (95% CI)
Male versus female	−5.35 (−10.7, 0.004)	*—*	−20.0 (−25.8, −14.2)^∗^
Age/10 years	3.17 (0.72, 5.62)^∗^	1.70 (−1.08, 4.49)	*—*
BMI weight (kg)/length m^2^	*—*	−0.58 (−1.24, 0.74)	−0.86 (−1.51, −0.21)^∗^
CABG versus valve surgery	*—*	3.63 (−1.25, 8.16)	*—*
ICU time/4 h	*—*	−0.24 (−0.56, 0.08)	*—*
*R* ^2^	0.07	0.09	0.33

VC: vital capacity, FEV_1_: forced expiratory volume in 1 second, IC: inspiratory capacity, BMI: body mass index, type of surgery (CABG: coronary artery bypass grafting, valve: aorta, mitralis, or tricuspidalis valve surgery), ICU: intensive care unit. ^∗^ *P* < 0.05, *B*: regression coefficient, CI: confidence interval, *R* ^2^: adjusted *R* square.

**Table 4:** The variables associated with decrease in postoperative lung volumes (in % of the preoperative value), (*n* = 107). Univariate analyses.

Independent variables	Dependent variables		
	VC	FEV_1_	IC
	*B* (95% CI)	*B* (95% CI)	*B* (95% CI)
Male versus female	—	—	7.24 (−0.25, 14.7)^†^
Diabetes	—	−9.22 (−18.2, −0.21)^∗^	—
Airflow obstruction		−9.72 (−19.9, 0.51)^†^	
Smoker versus no smoker	—	—	−15.5 (−26.8, −4.20)^∗^
Postoperative weight gain (hyperhydration), kg	—	−1.16 (−2.41, 0.09)^†^	—
ICU time/4 h	—	0.73 (0.54, 1.00)^∗^	—

VC: vital capacity, FEV_1_: forced expiratory volume in 1 second, IC: inspiratory capacity, airflow obstruction: defined as FEV_1_/VC or FVC < 70% or asthma, smoking (current smoker, smoker versus no smoker or nerver smoked), postoperative weight gain: relation between pre- and second postoperative day, ICU: intensive care unit. ^∗^ *P* < 0.05, ^†^ *P* = 0.05–0.10, *B*: regression coefficient, CI: confidence interval.
